# Factors predicting Behavior Management Problems during Initial Dental Examination in Children Aged 2 to 8 Years

**DOI:** 10.5005/jp-journals-10005-1397

**Published:** 2017-02-27

**Authors:** Arun Sharma, Dipanshu Kumar, Ashish Anand, Vipula Mittal, Aparna Singh, Nidhi Aggarwal

**Affiliations:** 1 Professor and Head, Department of Pedodontics and Preventive Dentistry, People’s College of Dental Sciences and Research Centre, People’s University, Bhopal, Madhya Pradesh, India; 2 Reader, Department of Pedodontics and Preventive Dentistry Institute of Dental Studies & Technologies, Ghaziabad, Uttar Pradesh, India; 3 Senior Lecturer, Department of Pedodontics and Preventive Dentistry Institute of Dental Studies & Technologies, Ghaziabad, Uttar Pradesh, India; 4 Consultant, Department of Prosthodontics, Dental Life Clinic, CGHS Dispensary, New Delhi, India; 5 Postgraduate Student, Department of Pedodontics and Preventive Dentistry Institute of Dental Studies & Technologies, Ghaziabad, Uttar Pradesh, India; 6 Professor and Head, Department of Pedodontics and Preventive Dentistry Institute of Dental Studies & Technologies, Ghaziabad, Uttar Pradesh, India

**Keywords:** Behavior, Dental anxiety, Management problems, Prediction.

## Abstract

**Aim:**

The aim of the present study was to identify the various background variables and its influence on behavior management problems (BMP) in children.

**Materials and methods:**

The study included 165 children aged 2 to 8 years. During the initial dental visit, an experienced operator obtained each child’s background variables from accompanying guardians using a standardized questionnaire. Children’s dental behavior was rated by Frankel behavior rating scale. The behavior was then analyzed in relation to the answers of the questionnaire, and a logistic regression model was used to determine the power of the variables, separately or combined, to predict BMP.

**Results:**

The logistic regression analysis considering differences in background variables between children with negative or positive behavior. Four variables turned out to be as predictors: Age, the guardian’s expectation of the child’s behavior at the dental examination, the child’s anxiety when meeting unfamiliar people, and the presence and absence of toothache.

**Conclusion:**

The present study concluded that by means of simple questionnaire BMP in children may be expected if one of these attributes is found.

**Clinical significance:**

Information on the origin of dental fear and uncooperative behavior in a child patient prior to treatment process may help the pediatric dentist plan appropriate behavior management and treatment strategy.

**How to cite this article:**

Sharma A, Kumar D, Anand A, Mittal V, Singh A, Aggarwal N. Factors predicting Behavior Management Problems during Initial Dental Examination in Children Aged 2 to 8 Years. Int J Clin Pediatr Dent 2017;10(1):5-9.

## INTRODUCTION

Despite the evolving nature of pediatric dentistry, a greater challenge facing the profession still remains: How to prevent or intercept dental fear/anxiety at the outset. Dental fear is a normal emotional reaction to one or more specific threatening stimuli in the dental situation, whereas the dental anxiety denotes a state of apprehension that something dreadful is going to happen in relation to dental treatment, and it is coupled with a sense of losing control.^1^ In practice differentiating dental anxiety, dental fear and phobia is complicated, and these terms are often used interchangeably in the literature. Moreover, the term “dental anxiety” is often used to include all different types of dental fears and phobias.^2^

Dental anxiety is a common problem which develops mostly in childhood and adolescence.^1,3^ Dental anxiety can have major implications for the child, dental team, and dental services. Providing treatment for the child patient with dental anxiety can be time-consuming, costly, and demanding for the clinician.^4,5^ Previous studies have shown that a majority of children with dental anxiety present behavior management problems (BMP) in dental treatment situations.^6^ Evaluating a child’s level of anxiety before pediatric treatment is the key, since this anxiety is closely related to their behavior during dental visits.^6^ Identifying and quantifying which factors trigger a situation of fear/anxiety is a basic requirement for controlling these factors to the largest extent possible and, therefore, reducing the child’s negative behavior during treatment.

The interaction of several variables, often suggested to be elements of a child’s behavior pattern, makes it difficult to study and analyze single elements. The objective of the science is to widen an understanding of the interpersonal social force that influences a patient’s behavior. Researchers have yet to determine which variables to target in order to improve their behaviors in a dental setting. No single assessment method or tool is completely accurate in predicting a child’s patients behavior for dental treatment, but awareness of the different types of background factors on child behavior may aid in predicting the behavior of pediatric patient.

Historically, research in the behavior aspects of children dentistry has focused on methods for altering the child’s behavior; however, the studies concerning the prediction of behavior of child in a dental clinic has received less consideration. Therefore, it is necessary to effectively predict children dental behavior, identify children at risk of BMP before such problems arise, and develop an appropriate management stratagem. Consequently, the present study was planned to explore and identify the various children background variables with a view to estimating their influence on BMP by means of a structured questionnaire.

## MATERIALS AND METHODS

This study included 165 children aged 2 to 8 years (90 boys and 75 girls) presenting for initial visit and seeking dental care. The criteria for including a child in the study were as follows: (1) Children aged 2 to 8 years; (2) child patients indicated for dental treatment including filling, tooth extraction, pulp therapy, pit and fissure sealants, and topical fluoride application; (3) the accompanying parent/guardian was one of the child’s primary caretakers; (4) the dentist had no previous connection with child or guardian; and (5) the accompanying person was able to understand and reply to our questions.

Children with dental emergencies, such as trauma, acute pulpitis, periapical abscess, and acute periapical periodontitis and the child with easily discernable mental limitations or communicative disorders were excluded from the study.

Upon arrival at the clinic, the guardians were informed of the aims of the study and could choose whether to take part in the study without affecting the dental care provided to child. An experienced operator elicited each child’s background variables from accompanying guardians using a standardized questionnaire. The questionnaire comprised of 16 questions, 8 of which had 3 to 5 alternative answers arranged as semantic scale and 8 of which had dichotomized answers (yes or no). The questions covered the child’s sociodemographic features (e.g., age, gender), previous dental and medical treatment experience, personality factors, health status, environmental conditions within or outside the family, and guardian’s attitude toward and experience with dental treatment.

The initial examination was evaluated in the following order: Step 1—enter treatment room; step 2—mirror in mouth; step 3—probe on fingernail and tooth surface; step 4—air blower on hand and in mouth; step 5—lie or sit in dental chair; and step 6—examination. The demands are gradually augmented in ascending order of relative magnitude as anxiety-provoking stimuli. Direct ratings of behavior were made by the Frankel behavior rating scale^7^ which was dichotomized into positive and negative behavior. The degree of acceptance was coded 1, 2, 3, or 4 according to the Frankel behavior rating scale. Positive behavior was defined as positive acceptance (rating 3 = positive; or 4 = definitely positive) whereas the negative behavior was defined as negative acceptance (rating 1 = definitely negative; or 2 = negative) at step 6.

The behavior was then analyzed in relation to the answers of the interviews, and a logistic regression model was used to determine the power of the variables, separately or combined, to predict BMP. Regression was performed on the data with complete questionnaires. Chi-square tests using exact methods to compute the statistical significance (p value) were used to compare possible predictors and uncooperative behavior.

**Table 1:** **Table 1:** Number and percentage of children with acceptance 1, 2, 3, and 4 at different steps

		*Negative acceptance*								*Positive acceptance*							
		*1*				*2*				*3*				*4*			
		*No.*		*%*		*No.*		*%*		*No.*		*%*		*No.*		*%*	
1. Enter treatment room		10		16.5		17		28		79		47.8		59		35.7	
2. Mirror in the mouth		18		10.9		25		15.1		72		43.7		50		30.3	
3. Probe on fingernail and tooth surface		20		12.1		24		14.5		69		41.8		52		31.6	
4. Air blower on hand and in mouth		16		9.7		25		15.1		72		43.6		52		31.6	
5. Lie or sit in the operating chair		12		7.2		30		18.2		66		40		57		34.6	
6. Examination		18		10.9		20		12.1		68		41.2		59		35.8	

## RESULTS

**Graph 1:**
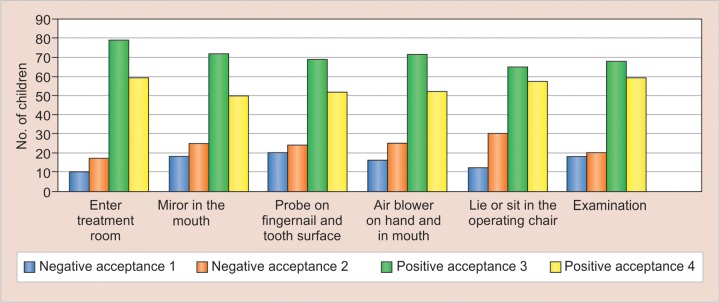
Number of children with acceptance 1, 2, 3, and 4 at different steps

**Graph 2:**
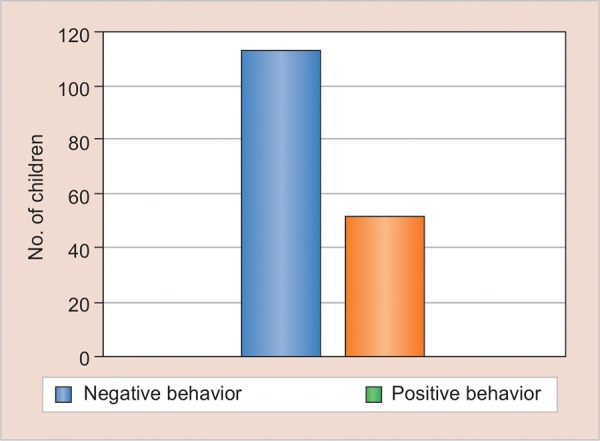
Guardian expectations of behavior

A total of 165 children aged 2 to 8 years were included in the study with a distribution of 85 males and 80 females. The mean age of the studied population was 4.2 years old with a standard deviation of 1.4 years. The numbers of children with acceptance 1, 2, 3, and 4 at different treatment steps are shown in Table 1 and Graph 1. The logistic regression analysis considering differences in background variables between children with negative or positive behavior at step 6 indicated four statistically significant variables as predictors: Age, the guardian’s expectation of the child’s behavior at the dental examination, the child’s anxiety when meeting unfamiliar people, and the presence and absence of toothache (Table 2). While 113 (68.4%) guardians expected positive dental behavior from their children, 52 (31.5%) expected negative behavior (Graph 2). More than one-third (60/165, 36.3%) of children were anxious around strangers, and 93 out of 165 (56.3%) of children had suffered toothache prior to the dental visit

## DISCUSSION

Before the 1960s, prevailing theories of children’s psychological development proposed that a child is a “tabula rasa,” or blank slate, i.e., imprinted upon by external forces which were considered to be the major formative factors in child behavior patterns.^8^ However, Stella Chess and Alexander Thomas after studying behavior patterns hypothesized that children are not “blank slates,” rather they have internal influences that lead to inherent individual differences in their reactions and motivations.^9^ A more comprehensive view may be that the child’s development and behavior are affected by a host of factors including individual physiology, temperament, and cognitive traits, as well as family and other environmental influences. Dental anxiety is likely to have multifactorial origins which are broadly divided into the internal and external origin. The external origin has been described as a simple conditioned phobia emerging from direct and indirect negative dental experience,^3,10^ and that internal origin can be characterized as a personality trait or endogenous anxiety, including factors related to the person.^11,12^

**Table 2:** **Table 2:** Variables found to predict children at risk of BMP during the initial dental visit

		*B*		*Wald*		*Sig*		*OR*	
Age		–0.775		18.146		<0.0001		0.313	
Guardian expectation		1.121		16.561		<0.001		3.063	
Anxiety around strangers		1.363		4.325		0.030		2.984	
Toothache		0.819		0.619		0.041		2.525	
Constant		0.925		0.989		0.328		2.589	

B: Regression coefficient; Sig: Significance correlation; OR: Odds ratio; BMP: Behavior management problems

The clinical pediatric dentist must be capable of effectively managing a child’s behavior and to accomplish this, there is need to discover what factors reduce anxiety and improve potentially negative behavior during a dental procedure. Through an understanding of child development and origin of dental fear and uncooperative behavior in a child patient prior to treatment process may help the pediatric dentist plan appropriate behavior management and treatment strategy. The response of a child patient to the demands of dental treatment is complex and determined by many factors. Child age/cognitive level, temperament/personality characteristics, anxiety and fear, reaction to the strangers, previous dental experience, and maternal dental anxiety influence a child’s reaction to the dental setting.^13^ Moreover, difficulties exist in assessing dental fear in younger children, and therefore its evaluation is largely based on interpretation of observed behavior in the dental situation using rating scales. Some authors have claimed that, because of limited emotional development of young children, questions should only be used with teenagers and adults.^14^ Since children exhibit a broad range of physical, intellectual, emotional, and social development, as well as a diversity of attitudes and temperament, the present study was designed to determine the influence of these factors derived from parental response on the prediction of behavioral responses during an initial dental appointment.

In the present study, logistic regression analysis identified four variables of the questionnaire that proved to be statistically significant predictors: The child’s age, the guardian’s expectation of the child’s behavior, the child’s anxiety when meeting strangers, and the presence of toothache. All these four factors proved to be important parameters, and many authors, as described below, have justified the predictors used in the present study.

Baier et al^15^ conclusions indicated that children under age 6 are more likely to exhibit negative behaviors, signifying that age may play a role both in behavior and anxiety in a dental setting. Hosey,^16^ in addition, suggested that the younger children are often afraid of the dental office, no matter what procedure they are facing. Moreover, Rud and Kisling^17^ stated that chronological age does not always correspond to level of mental development and stated that children who had reached a mental development corresponding to 29 months of age were able to cooperate in the dental situation. In the present study also, we found age to be one of the predictors which might be due to the fact that due to the limited intellectual development, the younger children were unable to be fully comprehended and realistically appraised the nature of dental treatment, or understood the explanations and instructions.

A guardian’s expectation of a negative reaction from a child was the second best predictor. The child’s behavior can be dependent on various factors like maturity, personality, and environmental factors, and the parents well aware of the effects of these background variables can very well predict the child’s ability to cope in the dental situation. These results are in accordance with studies carried out by Holst^18^ and Xia.^19^

Anxiety when meeting unfamiliar people is a normal, common part of a child’s developmental sequence that most children experience. Venham et al^20^ found that dental anxious children had stronger promotion of independence and weaker socialization training, resulting in anxiety when experiencing unknown people or situations. This background variable has been reported by various studies to be considered in predicting the child behavior.^18,19^ The present results are also compatible with the previously cited findings suggesting that not only the maturity and basic personality traits, but also the certain features of child rearing methods are significantly related to the children’s response to dental stress.

Children perceive and react to painful stimuli differently from each other, and studies have shown that children under the age of 4 are more sensitive to painful stimuli.^13^ Xia^19^ identified the presence of toothache as a risk factor for BMP; in addition, Poulton^21^ stated that symptomatic dental patient to be a predictor of early-onset dental fear. In a country like India, where health services have been usually oriented toward therapeutic care, toothache has been the main reason for seeking dental treatment. In the present study also, the presence of toothache was found to be a significant predictor. Our results showed that 58.7% of children had the history of toothache; moreover, 29% of the children presented for the first dental treatment because of toothache. Ferrari^22^ reported that fear of pain to be the most important predictor of dental anxiety. We believe that from a cognitive behavioral perspective, younger children with toothache to some degree anticipated aggravation of pain during the initial dental examination; furthermore, they are more likely to fear the consequences of pain sensations and more likely to avoid them, subsequently resulting in the negative behavior.

Versloot et al^23^ reported that the memory of previous dental experiences greatly influenced children behavior. In the present study, the majority of the children with previous dental experience displayed negative behavior, and it can be assumed that most of them were referred for BMP to specialized pediatric clinics. These findings suggest the importance of first dental visit which should be structured and organized in a playful and an enjoyable experience.

Based on this study, predictors for uncooperative behavior in the child were identified. The results of this investigation were established with a relatively simple questionnaire, and certain key questions asked prior to the initial dental examination may perhaps be helpful in assessing cooperative ability.

## CONCLUSION

Based on the results of this study, the following four factors were found to be predictive of children’s BMP in the dental clinic: The child’s age, the guardian’s expectation of the child’s behavior at the dental clinic, presence of anxiety around strangers, and the presence of toothache. Since there are many other variables that may influence behavior of children during dental appointment, further research using more appropriate methods are needed to clarify the role of these background variables in the BMP in children.
